# An Effect of Preparation Temperature on the Structure and Reactivity of Supported Ni–Cu Bimetallic Nanoparticles in Benzyl Phenyl Ether Hydrogenolysis

**DOI:** 10.1002/open.70287

**Published:** 2026-08-21

**Authors:** Raphaël Abolivier, Hans‐Georg Eckhardt, Jimmy Muldoon, Michael Fay, James A. Sullivan

**Affiliations:** ^1^ School of Chemistry University College Dublin Dublin Ireland; ^2^ Faculty of Engineering nmRC The University of Nottingham Nottingham UK

**Keywords:** bimetallic nanoparticles, dispersion, hydrogenolysis, lignin biorefining, reduction temperature

## Abstract

A set of four H‐ZSM‐5‐supported Ni–Cu‐containing materials with 5% Ni‐1% Cu loadings was prepared. The effect of using different reduction protocols (non‐reduced reference; H_2_ reduction at *T*_red._: 300; 400 and 500 °C) during the materials’ preparation was monitored to investigate the effect of this parameter on their microstructural features and their catalytic properties in a hydrogenolysis reaction. The variation of the reduction temperature has a direct effect on the composition and thus the separation of Ni atoms within the bimetallic nanoparticles and this in‐turn affects their reactivities for the hydrogenolysis of benzyl phenyl ether (a lignin model compound). Alloy Cu/Ni‐containing nanoparticles (confirmed by p‐XRD) prepared by reduction at 400 °C containing relatively more separated Ni atoms (observed using transmission electron microscopy ‐ energy dispersive x‐ray spectroscopy (TEM‐EDX)) were found to be more active in the reaction of interest than catalysts prepared using a reduction at 500 °C which had higher Ni contents (but lower Ni within particle separations).

## Graphical abstract

Ni is active in the hydrogenolysis reaction of interest, while Cu is inactive. However, catalysts containing bimetallic particles with fewer Ni atoms per particle are more active than bimetallic particles containing more Ni. The Ni loading in individual particles can be controlled by the preparation protocol.

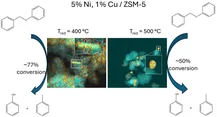

## 1 Introduction

Biorefining is expected to play a key role in the production of platform molecules for the chemical industry in the decades to come [1]. This relates to problems arising from the use of traditional petroleum‐based raw materials that are currently used to feed the chemical industry [2]. The efficient valorisation of renewable raw materials such as lignocellulose through biorefining processes will be of importance for the development of a more sustainable societal model [3]. Whilst numerous biorefining processes based on the valorisation of both cellulose and hemicellulose have already been, or are close to being, implemented on an industrial scales [4], the depolymerisation of lignin remains a difficult objective due to its complex and heterogeneous structure [5].

Lignin is the second most abundant natural biopolymer on earth and is composed of phenolic sub‐units [6], that, if extracted in a useful manner, could fuel numerous industries, from the production of pharmaceuticals [7] and plastics [8] to the sustainable generation of jet‐fuel [9]. Lignin is currently generated in large volumes through processes like pulping, but is mostly used as a low‐grade fuel for the generation of energy through combustion [10]. To exploit its full potential as a chemical precursor, lignin valorisation via depolymerisation needs to be further developed. Furthermore, given concerns about element criticality, such processes should rely on the use of cheap, efficient, and reliable catalysts [11].

Bimetallic nanoparticle catalysts have displayed interesting properties relating to improved robustness and often higher chemical reactivities with respect to their analogous monometallic catalysts [12]. Further studies will however, be needed to efficiently optimise both their composition and structures to promote specific reactions. Efforts must also be made for the use of abundant transition metals (TM) in these materials in lieu of the scarcer and more costly noble metals that are more commonly used [13].

**FIGURE 1:**
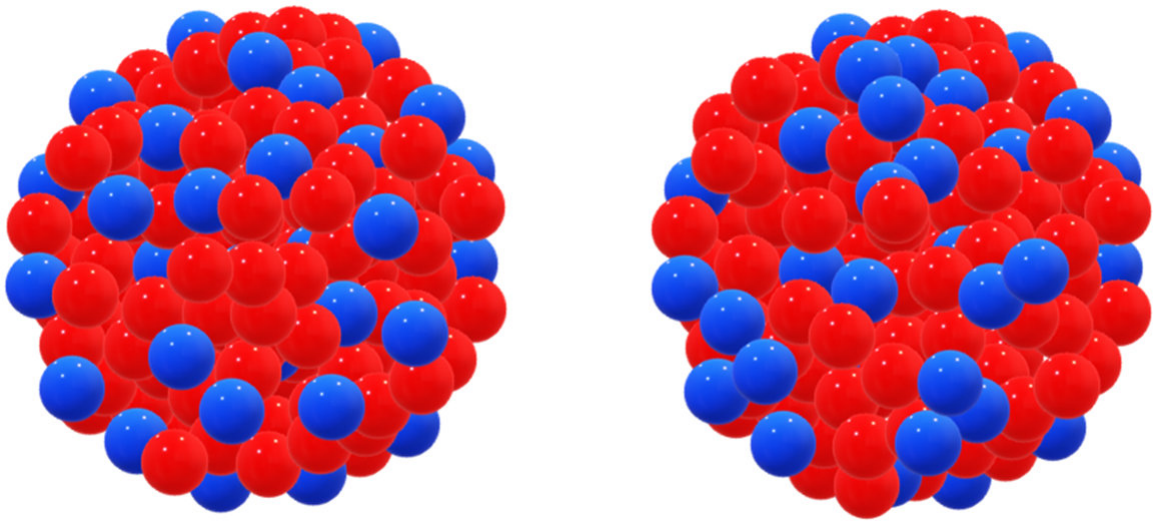
Representation of two bimetallic alloy nanoparticles with comparable compositions but different structural features.

For bimetallic materials, the structural arrangement of atoms of the different metals within the particles is a crucial factor in determining the material’s reactivity [14]. This feature is discussed here to describe the position of metal atoms within bimetallic particles. As an example, two bimetallic particles are shown in Figure 1, with red spheres representing the atoms of one metal and the blue spheres representing atoms of the other. It can be seen that both particles are alloys with similar compositions, but each has different structural features related to the different arrangements of atoms of the two metals within the particle structures.

Such differences within bimetallic particles can have important effects on the reactivity of these materials, due to the different extents of the resultant metal–metal interactions.

The placement of metal atoms within a given bimetallic particle is driven by particle and/or atom migration during the material preparation stage and is a challenging parameter to control using simple synthesis methods (e.g., impregnation) [15]. Advanced synthesis techniques (e.g., chemical vapor deposition‐based methods [16]) exist and, while these permit more control over these parameters [17], they are expensive and not suitable for large‐scale preparation of catalysts, e.g., as might be potentially used in biorefining facilities.

It has been shown that the arrangement of metal atoms within supported bimetallic particles prepared using an impregnation‐reduction technique can be modulated by varying the reduction protocol used [18]. Interestingly, variation of this feature in preparing supported bimetallic nanoparticles was also shown to impact the nature of the resulting bimetallic material. D’Souza et al. [19] showed that when using relatively high reduction temperatures (500 °C) for the preparation of a Pt–Co/C material, a homogeneous alloy structure was obtained, while when using relatively lower reduction temperatures (200 °C), core–shell (Co core and Pt shell) bimetallic structures were obtained. This change resulted from migration of Co atoms from the core to the surface (i.e., a modification of the Co atom structural arrangement within the particles) at the higher reduction temperatures. It was also proposed by Tang et al. [20] that the presence of Cu as a co‐catalyst in bimetallic structures could improve activity through a role as spillover sites in reaction involving hydrogen activation, allowing for a higher TOF over the nearby catalytic active site.

Whilst the modification of the reduction temperature may sometimes allow the preparation of a set of nanoparticles with pre‐determined compositions or structural features, its control can also hinder and/or favour, the ripening of nanoparticles into larger particles, modifying the nature of the bimetallic particles, and furthermore cause aggregation or segregation of atoms within individual particles. Different reduction protocols can be easily implemented at scale in the preparation of materials following simple impregnation processes.

The effect of using different reduction temperatures (300, 400 and 500 °C) for the preparation of bimetallic Ni–Cu/H‐ZSM‐5 supported catalysts was investigated in the present work. A reference, non‐reduced material was also investigated, yielding information on the formation mechanism of the bimetallic nanoparticles, following deposition via an excess‐impregnation method. The structures of the bimetallic particles of the prepared materials were characterised using p‐XRD and transmission electron microscopy (TEM)/TEM energy dispersive x‐ray spectroscopy (TEM‐EDX), yielding insights on the often‐overlooked effect of reduction temperature during the preparation of bimetallic catalysts.

This work sits within the continuity of results of similar reactions over bimetallic catalysts, e.g., those published by Cangiano et al. [21] who reported the formation of Ni–Cu solid‐solutions with heterogeneous Ni/Cu ratios upon reduction of the materials. The results presented here are also complementary to those of Pratama et al. [22] who recently studied the effects of Ni–Cu/ZSM‐5 bimetallic particle features in potential biorefining applications. In their work, materials with 1:1 Ni/Cu ratio (5% each) are investigated for the cleavage of benzyl phenyl ether, it was proposed that the presence of Cu in the material plays a key role through formation of bimetallic species with Ni. This was further investigated here using low Cu contents and focusing on monitoring the structural features of the prepared nanoparticles.

These catalysts were applied in the hydrogenolysis of benzyl phenyl ether (BPE) to toluene and phenol. BPE (1,2‐diphenoxyethane) is considered a lignin model compound because it contains the β‐O‐4 aryl ether linkage, which is the most abundant interunit linkage in native lignin. The differences in activity of the prepared materials confirmed that microstructural features impact a catalyst’s efficiency toward the promotion of this hydrogenolysis reaction. The catalysts were also characterised post‐reaction to investigate possible deactivation pathways along with an evaluation of the effects of harsh reaction conditions on the defined microstructural features.

## 2 Experimental

### 2.1 Catalyst Preparation

Commercially available NH_4_^+^‐ZSM‐5 was calcined in air at 550 °C for 3 h to prepare the H‐ZSM‐5 catalytic support. H‐ZSM‐5‐supported bimetallic Ni–Cu nanoparticle catalysts were then prepared using an excess‐impregnation synthesis. The appropriate amounts of nickel (II) acetate tetrahydrate and copper (II) acetate monohydrate (0.422 and 0.063 g, respectively, for the preparation of 2 g of 5% Ni‐1% Cu/ZSM‐5 material) were dissolved in an excess of deionised water. An appropriate mass of the H‐ZSM‐5 (1.8 g for the preparation of 2 g of material) was added to the solution. The obtained solution was stirred at R.T. (45 min) followed by an increase in temperature (to 90 °C) to allow solvent evaporation. The obtained powder was crushed and calcined in air at 500 °C for 3 h. The resulting material was then split into 4 equal masses. One of these was kept as such, while the other three were reduced in flows of 3% H_2_/Ar for 2 h at different temperatures (300, 400 and 500 °C). These samples were labelled using the reduction temperature used (i.e., 300, 400 and 500 °C, and as “Not Reduced”). All catalysts were used to promote the ether bond cleavage in benzyl phenyl ether, via hydrogenolysis (see below).

### 2.2 Catalyst Characterisation

All materials were characterised by power X‐ray diffraction (p‐XRD) on a Rigaku Miniflex 600 equipped with a source of Cu Kα radiation (*λ *= 1.54 Å). The scans were conducted in 2*θ* mode between 5° and 80°.

Samples for TEM and TEM‐EDX analysis were prepared by dispersion of a few mg of the materials in isopropanol. The suspensions were then sonicated for 15 min, and 10 μL was extracted and dropped onto Cu‐coated (for imaging) and Au‐coated (for EDX analysis) TEM grids. The solvent was then fully evaporated. The TEM images were recorded on a FEI Tecnai G2 20 Twin Microscope operated at 200 kV. The TEM‐EDX images were recorded on a JEOL 2100+ TEM equipped with an Oxford Instrument X‐MaxN 80 TLE EDX detector.

Thermogravimetric Analysis (TGA) measurements were performed under an air‐flow of 60 mL/min (with a balance flow of N_2_ of 60 mL/min) using a Q500 Thermogravimetric Analyser (TA Instruments). The temperature programme consisted of a 5 min. isotherm at room temperature, followed by a 5 °C/min temperature ramp up to 550 °C and another 5 min isotherm at this temperature.

### 2.3 Catalyst Application

All reactions were carried out in a high‐pressure, stirred, 300 mL bench‐top 4566 mini, Parr reactor. The temperature (300 °C) and stirring speed (800 rpm) were controlled using a 4848 Parr reactor controller. For all reactions, a suspension composed of the appropriate amount of benzyl phenyl ether (50 mg) and catalyst (25 mg) in isopropanol (50 mL) was loaded in the stainless‐steel vessel, which was subsequently mounted on the reactor and sealed. The reactor was flushed three times with H_2_ to remove O_2_ from the reactor, and the vessel was pressurised with H_2_ (g) (Initial Hydrogen Pressure, “IHP”: 8.0 bar) and heated to the required reaction temperature.

After the desired reaction time, i.e. 1 h, had elapsed, both the heating and stirring were switched off, and the vessel was rapidly cooled to room temperature using iced water. The reactor was depressurised, and the vessel disconnected. An aliquot of the liquid phase was collected directly from the vessel using a syringe equipped with a filter (to remove any catalyst particles) and loaded into a GC vial for analysis.

The identification of reaction products was carried out using a Waters GCT premier‐TOF coupled GC–MS apparatus. The mixture was analysed by GC–MS, using a HP‐5 ms GC column and a time‐of‐flight (TOF) detector. A Varian GC‐450 GC apparatus equipped with a HP‐5 column and FID detector was then used to quantify levels of formation of the different reaction products.

Following reaction, the catalysts used were collected using vacuum filtration and further characterised to investigate any structural changes that had taken place during the reaction and whether carbonaceous species had deposited on their surfaces during reactions.

The conversions, yields and selectivity of the hydrogenolysis reaction were calculated using the following equations:

$$\text{Conversion }\left(\%\right)=\frac{\text{Moles of substrate reacted}}{\text{Moles of substrate at}\;t_{0}}\times 100$$

$$\text{Yield }\left(\%\right)=\frac{\text{Moles of product generated}}{\text{Moles of substrate at}\;t_{0}}\times 100$$

$$\text{Select}.\text{hydrog. }\left(\%\right)=\frac{\text{Moles of hydrogenolysis products}}{2\times \text{Moles of converted substrate}}\times 100$$

The formation of carbonaceous deposits on the catalysts during reaction and partial over‐conversion of the desired reaction products to undesired molecules (the levels of formation of which have not been quantitatively measured) hindered the determination of a carbon balance for the reactions. However, satisfying selectivities toward the hydrogenolysis reaction for formation of the products of interest have been obtained in most reactions.

## 3 Results and Discussion

### 3.1 p‐XRD

**FIGURE 2:**
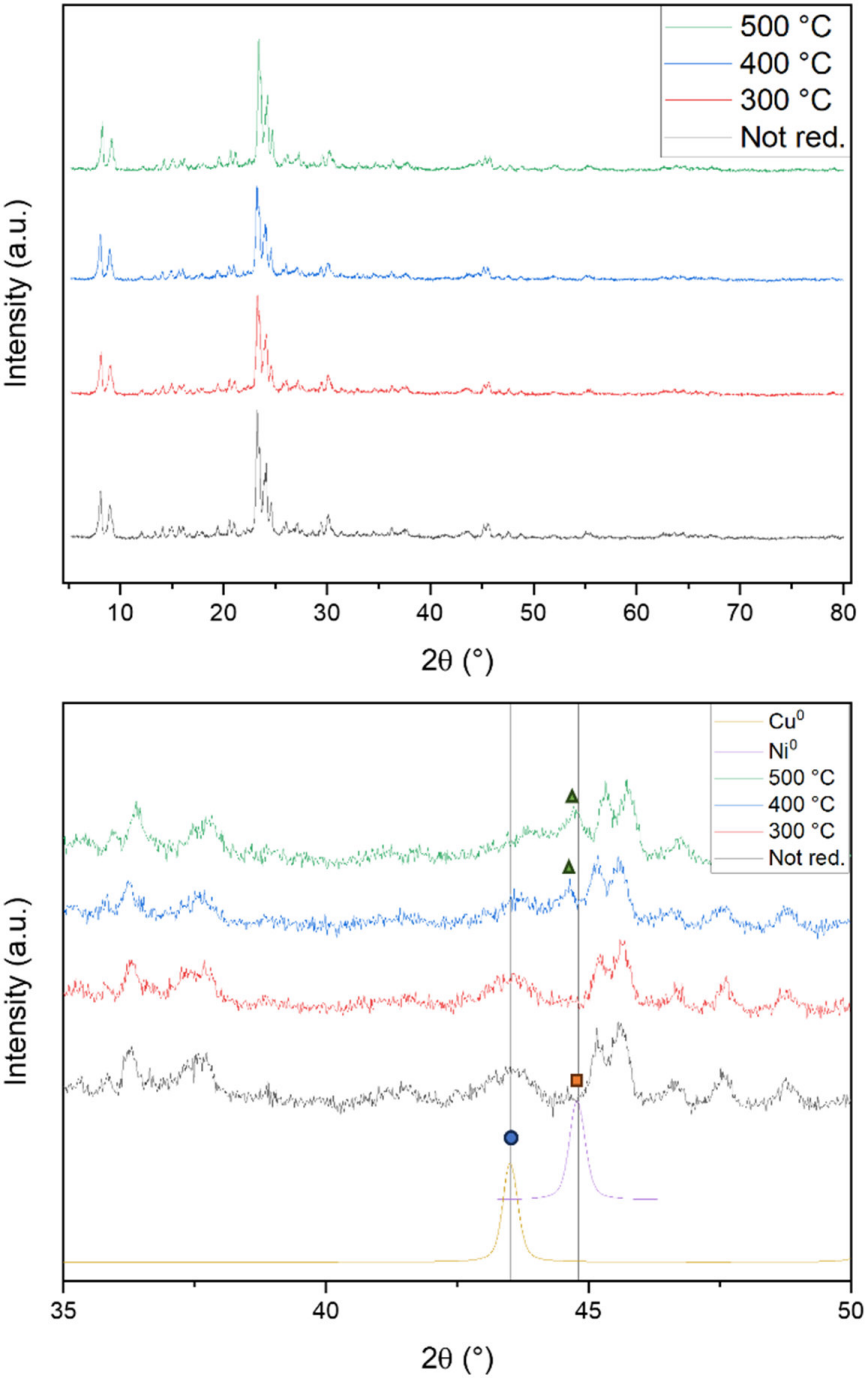
p‐XRD patterns of the 5% Ni‐1% Cu/ZSM‐5 materials. Overview profiles (upper plots; 2*θ*: 5°–80°). Expanded profiles on the region corresponding to the bimetallic structure peaks (lower plots; 2*θ*: 35°–60°, Cu^0^ °; Ni–Cu △; Ni^0^ □).

The p‐XRD patterns of the 5% Ni‐1% Cu/ZSM‐5 materials prepared at different reduction temperatures (300, 400, and 500 °C) are shown in Figure 2. The non‐reduced analogue of these materials was also analysed. The full profiles (2*θ*: 5°–80°) of these materials are shown in the upper plot, and an expanded (2*θ* between 35° and 60°) version of these profiles is shown in the lower plot (along with reference p‐XRD patterns of Ni^0^ and Cu^0^).

A set of diffraction peaks corresponding to those of a tetragonal MFI‐type structure (i.e., that of the H‐ZSM‐5 zeolitic support) was seen at diffraction angles of 2*θ*  =  8.2°, 9.1°, 13.9°, 23.4°, 24.2°, and 30.4° in the profiles of all materials. These relate to reflections from the (101), (111), (102), (051), (313), and (062) planes of the ZSM‐5 material [23]. This confirms that the metal loading protocol had no effect on the structure of the zeolite support.

No peaks suggesting the formation of a Cu/Ni alloy structure [24] (i.e., a single peak with a (111) reflection at a diffraction angle between that of Ni^0^ (111) (2*θ*: 44.7°) [25] and that of Cu^0^ (111) (2*θ*: 43.3°) [26]) were observed in the profiles of either the non‐reduced material or the material prepared at *T*_red._: 300 °C. This was expected for the former material, where metallic particles would not be expected (rather these should be Ni, and/or Cu oxides), but it suggest that a 300 °C reduction temperature is not sufficient for the reduction of these oxides to XRD‐detectable Ni–Cu nanoparticles. Literature suggests that CuO will be reduced at 300 °C, but that ZnO requires higher temperatures (although some reports suggests a catalytic effect of Cu on the reduction of ZnO [27, 28, 29].

A low‐intensity, very broad peak can however, be seen in the profiles of these two materials at a diffraction angle of 2*θ*: 43.2°. This could either relate to the formation of Cu^0^ species or to the presence of NiO nanoparticles [30]. No peaks corresponding to the presence of CuO or monometallic Ni^0^ structures could be seen in either profile.

However, a peak corresponding to diffraction by Ni–Cu alloy structures was observed in the p‐XRD patterns of materials reduced at both 400 and 500 °C. Interestingly, these do not peak at the same diffraction angle as one another (2*θ*: 44.5° and 44.6°, respectively). These peaks are between those expected for Cu and Ni (see above), and this suggests different Ni:Cu compositions within the formed nanoparticles depending on which reduction temperature had been used [31].

The use of the higher reduction temperature (500 °C) led to nanoparticles with a relatively higher Ni content, i.e., an alloy with a (111) diffraction peak at a higher angle (i.e., closer to the diffraction angle corresponding to that of Ni^0^). No peaks relating to monometallic Cu^0^ or Ni^0^ particles were evident in either of these profiles. This suggests that if monometallic particles of either Cu or Ni had formed on these materials, they are undetectable using p‐XRD. Overall, the reduction temperature was found to have a direct impact on the composition of the obtained nanoparticles in the materials. This is in line with the work by Oezaslan et al., who followed the evolution of Pd–Cu bimetallic alloy nanoparticles (supported on activated carbon) prepared at different reduction temperatures and have shown that the reduction temperatures affected the eventual bimetallic composition.

### 3.2 Electron Microscopy—TEM/TEM‐EDX

**FIGURE 3:**
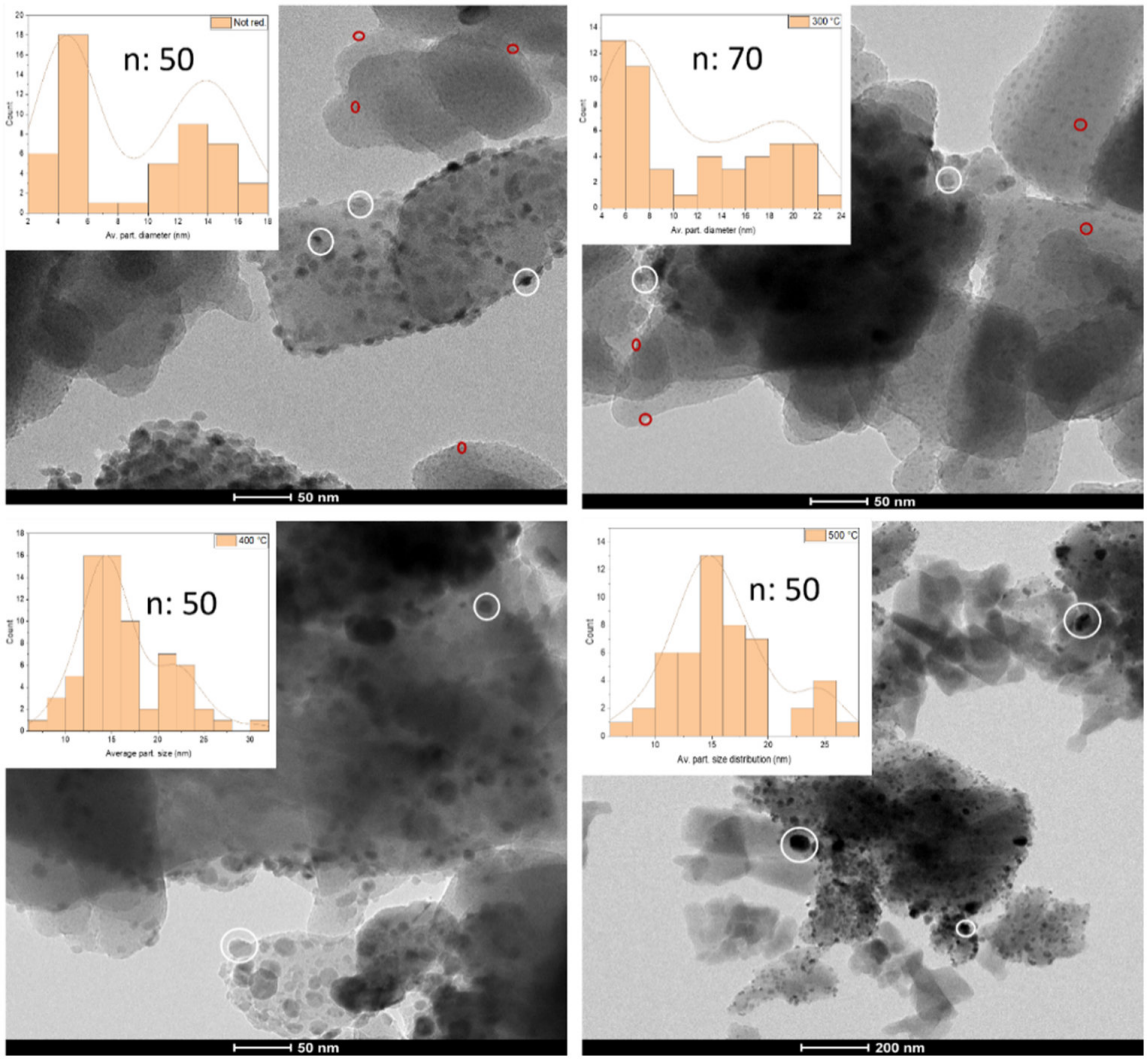
TEM images of the Ni–Cu/ZSM‐5 materials. Not reduced (top left); Reduced at 300 °C (top right), 400 °C (bottom left) and 500 °C (bottom right). Insets show histograms of Cu/Ni‐containing particle size distribution.

The TEM images of all materials in the 5% Ni‐1% Cu/ZSM‐5 series are shown in Figure 3. Histograms corresponding to the average Ni/Cu‐containing nanoparticle size in each of these images are shown as insets.

In each of the TEM images of the material that was not reduced (top left) and the material reduced at 300 °C (top right), two sets of nanoparticles with different average sizes can be observed. The first set is made of relatively small nanoparticles (<7 nm diameter on average—examples circled in red with the majority of particles being between 4 and 6 nm wide) for both samples, and the second set is composed of larger nanoparticles, with an average size of 14 nm for the non‐reduced material (with some particles measured at up to 18 nm) and of 19 nm for the material reduced at 300 °C (this set of particles is not homogeneous in size, with particles ranging from 12 to 22 nm; examples circled in white). On the non‐reduced sample, it can be assumed these are metal oxides (either mixed Ni/CuO or monometallic analogues).

The relatively small nanoparticles are not seen in the TEM images of the materials reduced at 400 °C (bottom left) or 500 °C (bottom right). This suggests that these species were removed (presumably through sintering) during the higher temperature reduction steps. This is an indication of a sintering phenomenon taking place during the reduction step. In the TEM images of the materials reduced at 400 and 500 °C, the observed nanoparticles are not formed in narrow size ranges. In both cases, two sets of particles (each, however, having a relatively narrow size distribution) are observed, centred at 14 and 22 nm for the material prepared at *T*_red._: 400 °C and centred at 15 and 24 nm for the material prepared at *T*_red._: 500 °C. Both sets of particles on both materials had an average particle size of ~16 nm. The observed evolution of measured nanoparticle sizes as a function of the reaction temperature is an indication that this step is crucial in the formation of nanoparticles with defined features. To further analyse this effect, TEM‐EDX measurements were performed.

TEM‐EDX images of both materials on which no bimetallic structures were detected by p‐XRD (i.e., the reference material and the material prepared at *T*_red._: 300 °C) are shown in Figure ‍4. Signals from Ni atoms are depicted in orange and those from Cu atoms in blue. Signals from oxygen atoms are also shown in green on the image of the *T*_red._: 300 °C material (highlighted to show the possible formation of metallic/bimetallic species on this sample).

**FIGURE 4:**
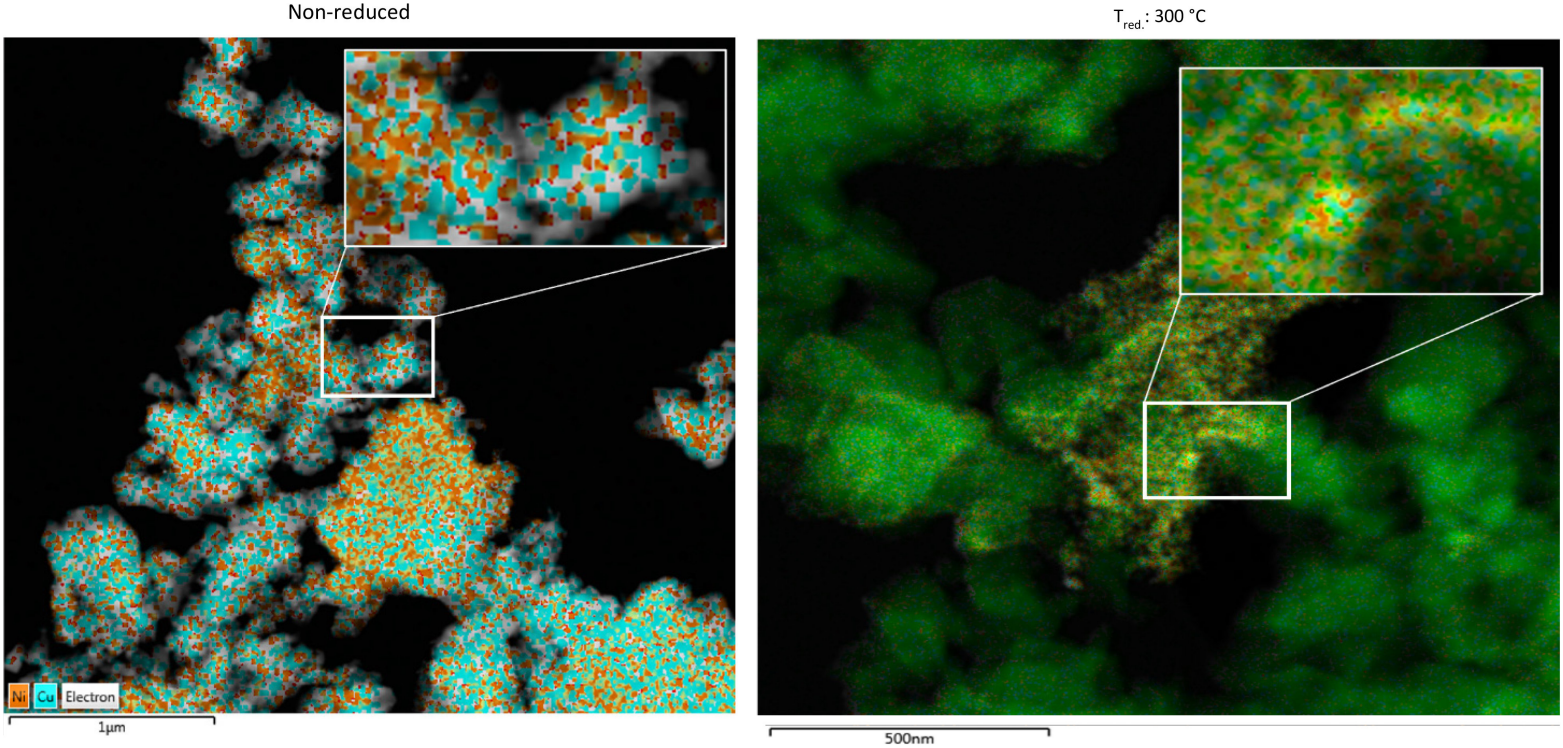
TEM‐EDX images of the reference, non‐reduced material (left) and of the material prepared at *T*_red._: 300 °C (right). EDX signals from Ni atoms are depicted in orange and those from Cu atoms in blue, O atoms are shown in green on the image on the right. An area with a representative nanoparticle is shown as insert in both images.

It can be seen that clearly separated Ni and Cu signals are observed in the image of the reference material (i.e., clearly orange or blue regions in the images—with relatively little yellow—which would indicate the presence of a bimetallic). This indicates that the alloyed bimetallic structures are formed during the reduction step of the synthesis process and not during the calcination step. This is in line with the observations made above regarding the evolution of the studied nanoparticle size following the reduction step.

In the image of the *T*_red._: 300 °C material, signals from oxygen atoms are largely detected from the H‐ZSM‐5 support; however, areas with metal‐only signals are also seen. This suggests the formation of small metallic nanoparticles on this material. The TPR data cited above suggests these may be Cu(0) particles, with the ZnO materials remaining unreduced following the 300 °C treatment. Interestingly, whilst areas with clear signals from Ni and Cu atoms are observed in the image of the *T*_red._: 300 °C material, areas appearing in yellow (representing zones where signals from both metals were detected, i.e., an overlay of both blue and orange) are noticed in the vicinity of Ni atom clusters. This indicates that some alloy structures may have formed as a minor species at this reduction temperature. This highlights the differences in sensitivities between this technique and p‐XRD, as these were not noted above (Figure 2). The location of these structures (i.e., associated with Ni atom clusters) suggests that, following this reduction temperature, the bimetallic nanoparticles were formed by movement of Cu atoms onto NiO nanoparticles.

**FIGURE 5:**
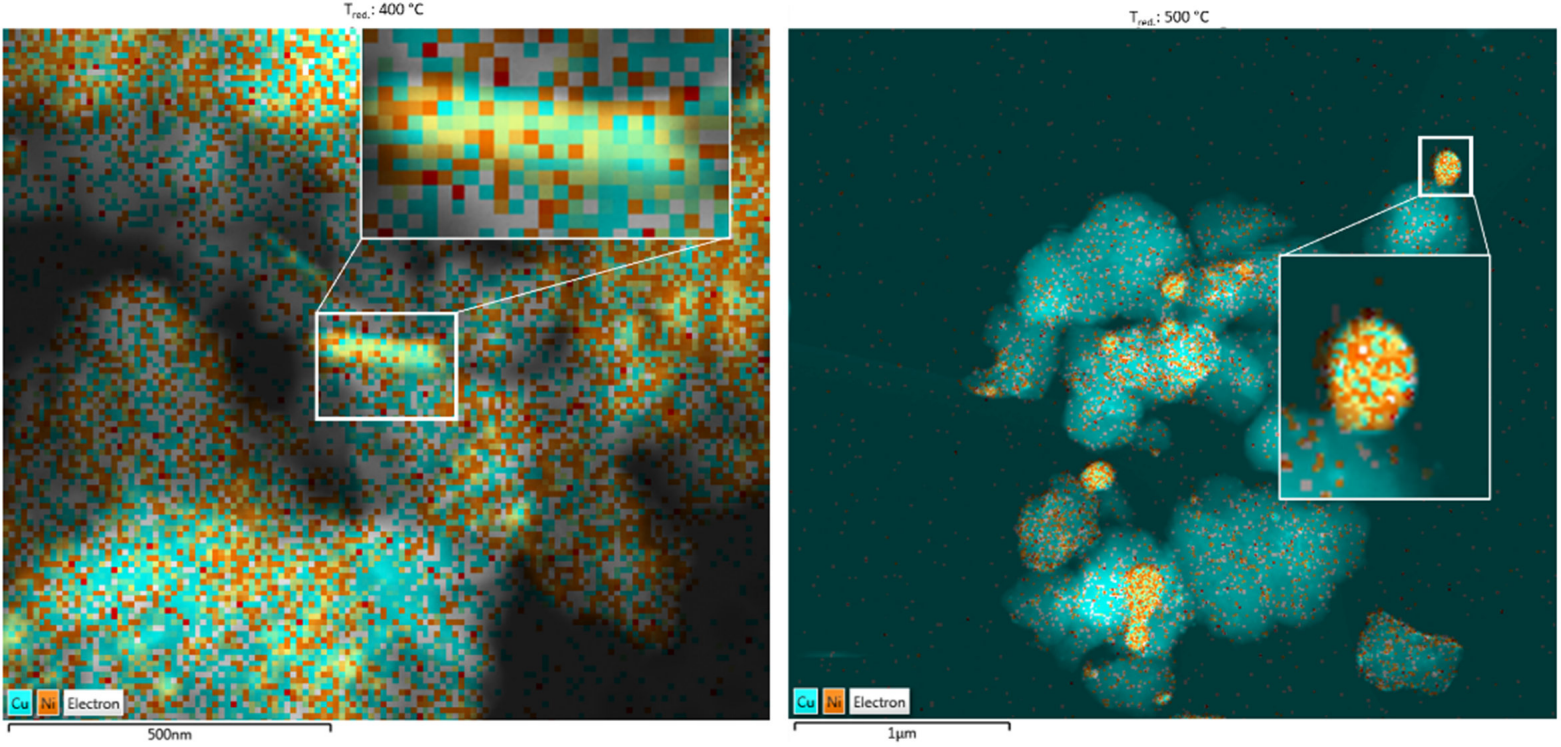
TEM‐EDX images of the materials prepared at *T*_red._: 400 °C (left) and *T*_red._: 500 °C (right). Ni atoms are depicted in orange and Cu atoms in blue. An area with a representative nanoparticle is shown as insert in both images.

Both materials prepared following higher temperature reduction (i.e., *T*_red._: 400 °C and *T*_red._: 500 °C), and which were confirmed by p‐XRD to contain alloy nanoparticles, were also further characterised using the TEM‐EDX technique to collect information on the location of Ni and Cu atoms within the catalysts. These TEM‐EDX images are shown in Figure 5.

It can be seen in both images that the nanoparticles are composed of both Ni and Cu (confirming the formation of alloy nanoparticles and in line with the p‐XRD observation made above). In both, there appear to be areas of relatively higher Cu and Ni concentrations. Interestingly (see insets), the alloyed nanoparticles were not found to have the same structures as one another. Nanoparticles on the material prepared at a higher reduction temperature (i.e., *T*_red._: 500 °C) have more Ni atoms (as also suggested by p‐XRD), and these Ni atoms are also more aggregated within the particles. Conversely, the signals from Ni atoms within the nanoparticles on the material prepared at the relatively lower reduction temperature (i.e., *T*_red._: 400 °C), which individually show fewer Ni signals, also show signals that are more separated from one another (which is to be expected given the relatively lower Ni loadings in each particle).

These observations, coupled with those made from the p‐XRD analysis above, suggest that a higher Ni content in the individual nanoparticles results in lower levels of separation of the Ni atom‐rich domains from each other within the nanoparticles.

The effect of the catalyst reduction temperature on the structure of ZrO_2_‐supported Ni–Cu nanoparticles was also noted by Wolfbeisser et al. [32]. They reported that Cu atoms tend to easily aggregate on bimetallic nanoparticle surfaces following preparation using relatively low reduction temperatures, but that an increase in reduction temperature led to Ni atom segregation on the material surface and this feature was independent of the nominal loadings of either metal. This difference in behaviour could be attributed to different surface energies of the atoms of the two different metals. This is also supported by similar findings from Luan et al. [33] in a theoretical study looking at unsupported Ni–Cu nanoparticle structural feature evolution following different reduction temperatures.

Overall, TEM‐EDX analysis showed the formation of alloys for all reduced materials;, however, as a minor species for the material prepared at *T*_red._ = 300 °C, the lowest reduction temperature, whilst no clear metal aggregation could be observed for the reference, non‐reduced material. The reduction temperature used for the material preparation also influenced the levels (and subsequently the arrangement) of the metals in the alloy nanoparticles. These results, along with those discussed above regarding nanoparticle size evolution using different reduction temperatures, could be of interest in the design of catalysts with defined features using a simple preparation protocol.

### 3.3 Catalytic Activity

**FIGURE 6:**
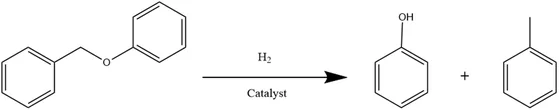
Reaction scheme for the catalysed hydrogenolysis of benzyl phenyl ether to toluene and phenol.

The Ni–Cu/ZSM‐5 bimetallic catalysts were used to promote the hydrogenolysis of benzyl phenyl ether (BPE) to toluene and phenol (see Figure 6). The reaction mechanism and roles of metallic nanoparticles over both monometallic [34] and Ni‍–‍Cubimetallic [35] H‐ZSM‐5‐supported catalysts have already been reported and discussed in more detail in our previously published work regarding similar materials. Monometallic Ni‐based catalysts were shown to be relatively efficient for the conversion of BPE at high metal loadings (>10%) while monometallic Cu‐based materials were generally unreactive. Preliminary tests using monometallic catalysts clearly indicated that Cu alone did not promote the reaction whilst Ni alone could. Over bimetallic Ni–Cu/ZSM‐5 catalysts prepared with varied metal ratios, Ni atoms were shown to be the active reaction sites for the hydrogenolysis reaction, with Cu atoms playing a key role as cocatalyst. In all cases, bimetallic materials were more efficient than Ni‐based analogues. The role of Cu in these bimetallic structures was further investigated here via the prism of the effect of reduction temperature on low Cu‐content catalysts. The investigated hypothesis is that Ni–Cu interactions play a key role in the reactivity of the bimetallic materials in the studied reaction. Based on the observation discussed above regarding the different features of the prepared catalysts, it is expected that different reactivity over these materials having comparable metal loadings would provide significant empirical insight onto the role of “active metal”–“host metal” interactions in the observed catalysis.

The conversion of BPE to toluene and phenol at 300 °C was monitored over all catalysts in the series, and results are presented in Table 1. The conversion of BPE in the absence of a catalyst was 7.2% with low selectivity to both of the expected compounds (<10%), indicating degradation of the substrate and/or conversion to undesired products.

**TABLE 1:** Investigation of the 5% Ni‐1% Cu/ZSM‐5 catalysts prepared at different *T*_red._ for the conversion of BPE to toluene and phenol.

Catalyst	BPE conversion, %	Toluene yield, %	Phenol yield, %	Selec. hydrog., %
Not reduced	32.5	24.1	22.0	71.0
300 °C	40.7	40.7	36.1	94.4
400 °C	77.0	76.2	64.5	91.4
500 °C	49.5	47.5	42.4	90.9

*Note:* Reaction conditions: BPE (50 mg); catalyst (25 mg); isopropanol (50 mL); *T*: 300 °C; IHP: 8.0 bar; *t*: 1 h.

Relatively low BPE conversions were obtained over the reference, non‐reduced material (32.5%) and over the material reduced at 300 °C (40.7%).

In the former case, this was attributed to the lack of metallic Ni domains on this material. Relatively low yields of toluene and phenol were obtained, with values of 24.1% and 22.0%, respectively (for an overall selectivity toward the hydrogenolysis reaction of 71.0%); this was attributed to the partial conversion of BPE to benzylphenols, following reaction of the substrate over the acid sites of the zeolite support [36].

The relatively higher reactivity of the latter catalyst (40.7% conversion), along with the higher selectivity toward both products of interest, could be linked with the presence of low levels of alloyed bimetallic nanoparticles as seen by TEM‐EDX. This suggests that even low levels of bimetallic nanoparticles improve the reactivity of these catalysts for reaction of interest. It is also possible that the nature of the nanoparticles has been modified under reaction conditions, this is further discussed below.

Higher conversion and selectivity to the desired hydrogenolysis reaction products were obtained over the other two catalysts. A significantly higher BPE conversion was noted over the catalyst prepared at *T*_red._: 400 °C (77.0%) than over the catalyst prepared at *T*_red._: 500 °C (49.5%). Whilst Ni surfaces are the active species for the promotion of the reaction [37], interestingly, the material on which the nanoparticles have the higher Ni content (i.e., the material prepared at *T*_red._: 500 °C, as seen by p‐XRD, and confirmed by EDX) is not the most active for the reaction. This suggests that the material with a lower Ni content, with a resultant higher separation of the Ni atoms within individual nanoparticles (as seen in TEM‐EDX images), is the reason for the higher reactivity. It is proposed that this difference in reactivity arises from the difference in nanoparticle structure and composition, and specifically from the levels of Cu atoms in particles, which led to resultant changes in separation between Ni species within the individual nanoparticles on these materials.

Interestingly, a very high selectivity toward aromatic products was also noted here, with >90.0% hydrogenolysis products generated over all the reduced catalysts. This is not usually observed in literature, where cyclic compounds are often formed over comparable bimetallic catalysts in comparable reactions. Chen et al. [38] and Guo et al. [39] used Ni–Co‐based catalysts for the conversion of diphenyl ether, producing cyclohexene and cyclohexanol (along with diclyclohexyl ether) as the main reaction products in both cases. Zhang et al. [40] also studied the conversion of benzyl phenyl ether over Ni–Ru/ZrO_2_ and obtained similar results. Wu et al. [41] used Ni–Cu/Al_2_O_3_ catalysts for the conversion of benzyl phenyl ether, with cyclohexanol formed as one of the main reaction products instead of phenol (as is the case in the present study). This suggests the interest of Ni–Cu/ZSM‐5 materials with defined features in the potential production of value‐added compounds within biorefinery facilities.

These differences in activity suggest that further attention should be given to catalytic material synthesis methods, specifically regarding their microstructural features. It was shown that relatively small differences in preparation, i.e., here the reduction temperature used, can lead to important differences in structure and subsequently in activity for well‐known reactions. These results suggest that optimisation of often overlooked synthesis parameters can lead to important improvements in performance in industrially relevant processes.

### 3.4 Post‐Reaction Characterisation

**FIGURE 7:**
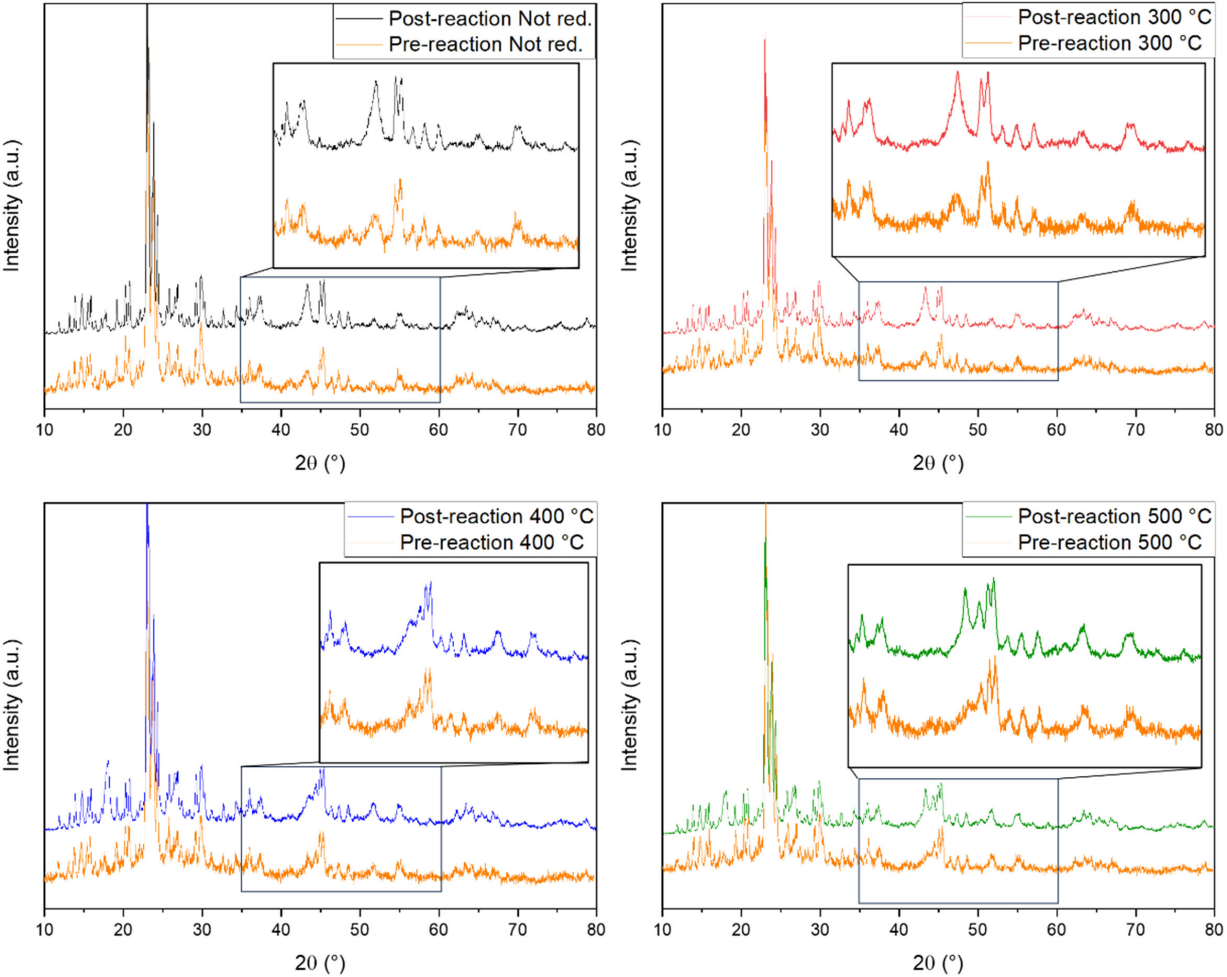
Post‐reaction p‐XRD patterns of the catalysts in the Ni–Cu/ZSM‐5 series (2*θ*: 5°–80°). The insets display the 2*θ*: 30°–65° region.

Following reaction, the catalysts were collected by filtration and characterised to investigate possible evolution of the material’s properties during reaction. The p‐XRD profiles of the pre‐ and post‐reaction catalysts are compared in Figure 7. The 2*θ* angle range containing the peaks related to the alloy structures (35°‍–60°)‍ in all profiles is shown in the insets.

Interestingly, in the profiles of the two less reactive materials (i.e., the reference, non‐reduced material and the material prepared at *T*_red._: 300 °C), the broad peak noted at 2*θ*: 43.2° increased significantly in intensity. In the pattern of the former material, no shift in position of this peak was noted, indicating that small particles sintered without any noticeable effect on their composition. However, a slight shift toward a higher diffraction angle was noted in the profile of the latter material (i.e., *T*_red._: 300 °C), suggesting that, during the reaction, relatively higher levels of alloyed Ni–Cu may have formed on this material following interaction between the catalyst and gaseous H_2_ (recall the reactions are at 300 °C, the same temperatures at which the initial material was reduced during preparation).

A very low intensity peak in the pattern of the pre‐reaction material prepared at *T*_red._: 500 °C (observed around 2*θ*: 43.4°) significantly increased in intensity and slightly shifted to a lower diffraction angle (i.e., closer to that of Cu^0^). This indicates an appreciable amount of Cu uptake into pre‐existing bimetallic nanoparticles during the reaction. Conversely, the p‐XRD profiles of the pre‐ and post‐reaction material prepared at *T*_red._: 400 °C are similar to one another and do not indicate any change during the reaction.

TEM images of the post‐reaction catalysts are shown in Figure ‍8. As above, the histograms shown as insets show nanoparticle size analysis.

**FIGURE 8:**
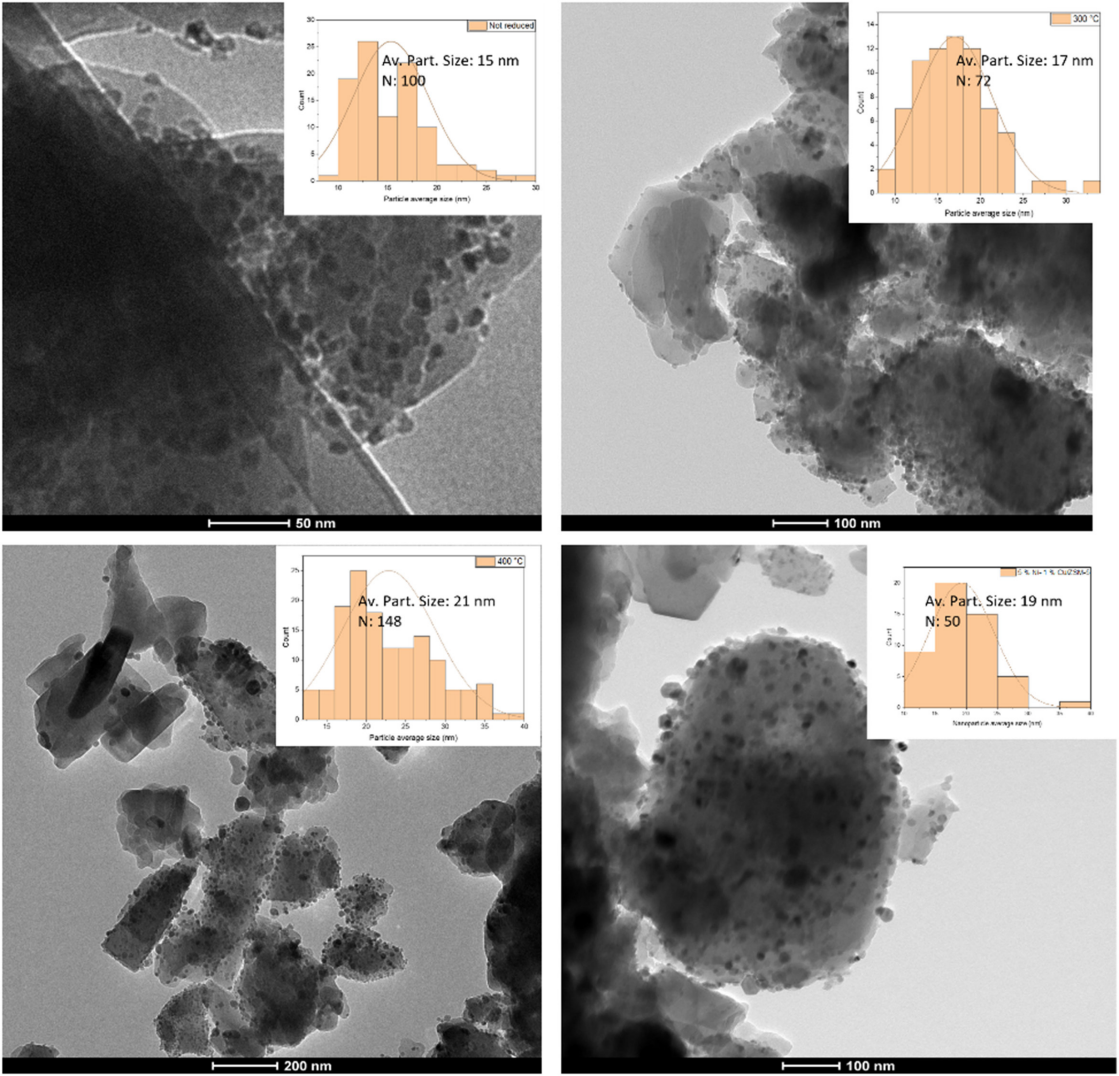
TEM images of the 5% Ni/1% Cu‐ZSM‐5 series. Not reduced (top left); Reduced at *T*_red._: 300 °C (top right); *T*_red._: 400 °C (bottom right); and *T*_red._: 500 °C (bottom left). Insets show the corresponding particle size distribution histograms.

Spherical nanoparticles are observed in the TEM images of all the materials. Interestingly, the nanoparticles are seen in a narrow size distribution range for all materials. Recall, a bimodal distribution was noted, i.e., a set of smaller nanoparticles and a set of relatively larger nanoparticles in the images of both the non‐reduced and the *T*_red._: 300 °C materials in the pre‐reaction TEM images of these materials. These small nanoparticles are not noted in TEM images of either of these materials post‐reaction. This suggests that the small nanoparticles sintered (possibly following reduction from oxide to metallic state) during reaction.

**FIGURE 9:**
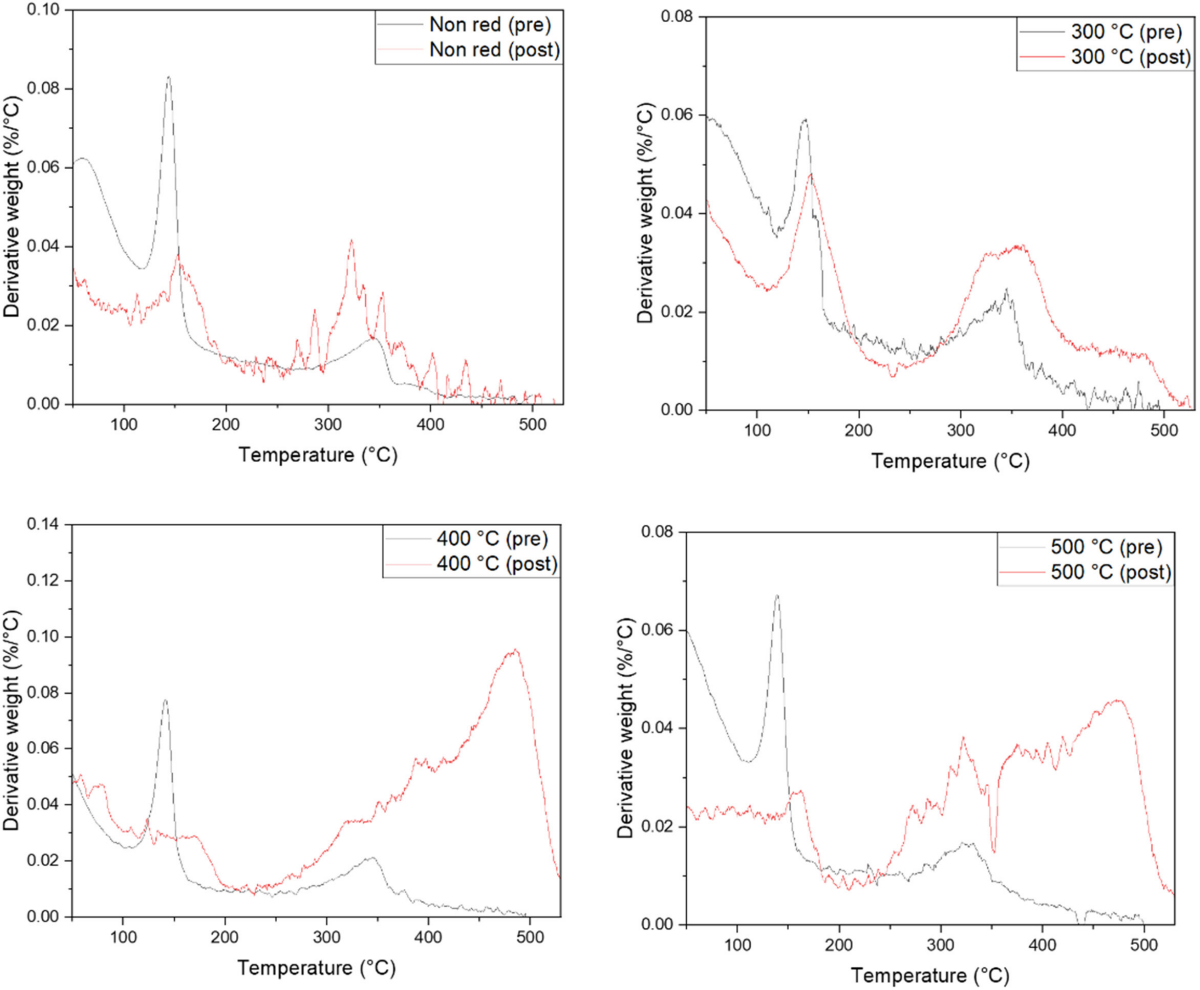
DTGA profiles for all materials pre‐(black profiles) and post‐(red profiles) reaction, non‐reduced material (top left), *T*_red._: 300 °C (top right); *T*_red._: 400 °C (bottom left) and *T*_red._: 500 °C (bottom right).

For the non‐reduced material, an increase in average particle size from 9 to 15 nm is seen, while for the material reduced at 300 °C, the increase is from 11 to 17 nm. For the material prepared at *T*_red._: 400 °C, the average nanoparticle size increased from 16 to 21 nm (with, as noted from XRD, an increase in Cu content), and for the material prepared at *T*_red._: 500 °C, the increase was from 16 to 19 nm (with, as noted above, an increase in Cu loading). To investigate the possible deposition of carbonaceous materials during reaction, TGA measurements were performed over all catalysts both pre‐ and post‐reaction. The results are shown in Figure 9.

These results confirm that significant amounts of metallic species remain on the surface of all materials following reaction; however, we have not analysed the reaction mixture for Ni or Cu species to confirm leaching. Furthermore, these changes suggest the reactivity of these materials in a catalyst recycling experiment would not be the same as that of the fresh material (although we have not carried out these experiments).

All pre‐reaction materials show a weight loss event at relatively low temperature (around 150 °C), attributed to the desorption of water from the porous zeolitic support [42]. As the post‐reaction materials were dried under air following reaction, this peak is significantly lower in intensity in the corresponding post‐reaction patterns. Over the non‐reduced material, low levels of carbonaceous material were deposited during reaction as seen by the relatively low mass loss noted at high temperature in the DTGA profile of the post‐reaction catalyst (at temperatures where such material would be combusted from the surface). This reflects the low reactivity of the material with low levels of substrate conversion, in turn leading to low levels of carbonaceous deposits.

Two peaks relating to high temperature mass loss events (i.e., the removal of a carbonaceous residue) can be seen in the profile of the post‐reaction material prepared at *T*_red._: 300 °C; these are also of relatively low magnitudes, again reflecting a relatively low reactivity. In the DTGA profiles of the post‐reaction materials prepared at *T*_red._: 400 °C and *T*_red._: 500 °C, the removal of far larger amounts of carbonaceous deposits was noted, see the significant mass loss events above 300 °C [43].

These two catalysts were the most reactive for the studied reaction with the material prepared at a reduction temperature of 400 °C being both the most efficient promoter of the reaction and the material upon which the larger amount of char was formed. Overall, no important evolution of the crystalline structure of the supported alloy nanoparticles was seen, except in the material prepared at *T*_red._: 500 °C, suggesting that microstructural features influence both the activity of the materials and their stability under harsh reaction conditions. Significant amounts of carbonaceous deposits were only detected on the materials prepared at *T*_red._: 400 and 500 °C (i.e., the most reactive materials), and a reasonable stability of the particle morphology and size was concluded from post‐reaction TEM images.

## 4 Conclusions

This work has studied the effect of reduction temperature on the properties of a set of ZSM‐5‐supported bimetallic 5%Ni‐1%Cu/ZSM‐5 materials prepared by excess solution impregnation followed by drying, calcination, and reduction steps.

The presence of high levels of metallic alloy nanoparticles was confirmed on the two materials prepared at *T*_red._: 400 and 500 °C by XRD and TSM/EDX. However, individually, these nanoparticles had different compositions (in terms of their Ni:Cu ratios). A higher reduction temperature triggered more Ni mobility and the formation of larger bimetallic particles with a proportionately higher Ni content. Recall, Ni is the active component promoting this reaction.

As a result of this differing Ni loading in individual particles, the Ni distribution within the alloyed nanoparticles of the two active materials was also different to one another, with the levels of Ni atoms being lower within the nanoparticles of the material prepared at the lower reduction temperature (and thus the dispersion of Ni being higher). Comparison with the reference material suggested that both diffusion of metallic particles on the support’s surface and aggregation of metal atoms within the formed nanoparticles had a role in the eventual microstructures of the supported nanoparticles. Both phenomena were directly correlated to the reduction temperature used during preparation.

These two catalysts showed significant reactivity with high conversions and selectivities to the products of interest. The catalyst containing nanoparticles with the higher dispersion of Ni atoms within individual nanoparticles (i.e., the material prepared at *T*_red._: 400 °C) was the most reactive for the reaction, with a 77.0% substrate conversion (vs. 49.5% over the material prepared at *T*_red._: 500 °C). This is unexpected because the active component in the promotion of this reaction is Ni (as shown in previous work), and the bimetallic nanoparticles on this material had lower Ni contents than those of the material reduced at 500 °C.

These two materials were found to be relatively stable under these reaction conditions, with low levels of nanoparticle sintering observed following reaction. Carbonaceous deposits were formed in relatively large amounts on both reactive materials.

## Funding

This work was supported by the Science Foundation Ireland (16/RC/3889), Engineering and Physical Sciences Research Council (EP/L022494/1 and EP/W006413/1), and the European Union HORIZON TMA MSCA Staff Exchanges (HORIZON‐MSCA‐2021‐SE‐01), grant agreement no 101086071, project name “CUPOLA — Carbon‐neutral pathways of recycling marine plastic waste”.

## Conflicts of Interest

The authors declare no conflicts of interest.

## Data Availability

The data that support the findings of this study are available from the corresponding author upon reasonable request.
